# Development of the VIGS System in the Dioecious Plant *Silene latifolia*

**DOI:** 10.3390/ijms20051031

**Published:** 2019-02-27

**Authors:** Naoko Fujita, Yusuke Kazama, Noriko Yamagishi, Kyoko Watanabe, Saki Ando, Hiroyuki Tsuji, Shigeyuki Kawano, Nobuyuki Yoshikawa, Ken Komatsu

**Affiliations:** 1 Kihara Institute for Biological Research (KIBR), Yokohama City University, 641-12 Maioka, Totsuka, Yokohama, Kanagawa 244-0813, Japan; tsujih@yokohama-cu.ac.jp; 2 Laboratory of Plant Pathology, Faculty of Agriculture, Tokyo University of Agriculture and Technology (TUAT), 3-5-8 Saiwaicho, Fuchu, Tokyo 183-8509, Japan; s185614v@st.go.tuat.ac.jp (S.A.); akomatsu@cc.tuat.ac.jp (K.K.); 3 RIKEN Nishina Center, 2-1 Hirosawa, Wako, Saitama 351-0198, Japan; ykaze@riken.jp; 4 Plant Pathology Laboratory, Faculty of Agriculture, Iwate University, Morioka, Iwate 020-8550, Japan; nyamagi@iwate-u.ac.jp (N.Y.); yoshikawa@iwate-u.ac.jp (N.Y.); 5 Mycology and Metabolic Diversity Research Center, Tamagawa University Research Institute, Machida 194-8610, Japan; wkyoko@agr.tamagawa.ac.jp; 6 Future Center Initiative, The University of Tokyo, FC503, 178-4-4, Wakashiba, Kashiwa, Chiba 277-0871, Japan; kawano@edu.k.u-tokyo.ac.jp

**Keywords:** dioecious plant, XY chromosomes, ALSV, *Microbotryum*

## Abstract

(1) Background: *Silene latifolia* is a dioecious plant, whose sex is determined by XY-type sex chromosomes. *Microbotryum lychnidis-dioicae* is a smut fungus that infects *S. latifolia* plants and causes masculinization in female flowers, as if *Microbotryum* were acting as a sex-determining gene. Recent large-scale sequencing efforts have promised to provide candidate genes that are involved in the sex determination machinery in plants. These candidate genes are to be analyzed for functional characterization. A virus vector can be a tool for functional gene analyses; (2) Methods: To develop a viral vector system in *S. latifolia* plants, we selected *Apple latent spherical virus* (ALSV) as an appropriate virus vector that has a wide host range; (3) Results: Following the optimization of the ALSV inoculation method, *S. latifolia* plants were infected with ALSV at high rates in the upper leaves. In situ hybridization analysis revealed that ALSV can migrate into the flower meristems in *S. latifolia* plants. Successful VIGS (virus-induced gene silencing) in *S. latifolia* plants was demonstrated with knockdown of the phytoene desaturase gene. Finally, the developed method was applied to floral organ genes to evaluate its usability in flowers; (4) Conclusion: The developed system enables functional gene analyses in *S. latifolia* plants, which can unveil gene functions and networks of *S. latifolia* plants, such as the mechanisms of sex determination and fungal-induced masculinization.

## 1. Introduction

*Silene latifolia* is a dioecious plant model system, where sex is determined by the XY sex chromosomes. This plant has been applied for various studies, including those on plant sex chromosome evolution, sex determination, dosage compensation, and DNA methylation [1,2,3,4,5,6]. A key factor in each of these aspects may be the sex-determining genes. Previous studies suggested that there are two sex-determining genes located on the Y chromosome, designated as stamen-promoting function (SPF) and gynoecium-suppressing function (GSF) [7]. Since the sex of *S. latifolia* is insensitive to exogenous plant hormones, it is thought that the presence of SPF and GSF are indispensable for the expression of the male phenotype, albeit with the one exception of fungal-induced masculinization. *S. latifolia* female flowers develop stamens when an anther smut fungus, *Microbotryum lychnidis-dioicae*, infects female plants, as if *Microbotryum* acts as an SPF (shown in the cover figure). Although the detailed molecular mechanisms of this phenomenon have largely remained a mystery, the stamens produced by *Microbotryum* infection are true stamens that have originated from the ordinary suppressed stamen primordia in female flowers [8].

Since the discovery of sex chromosomes in *S. latifolia* plants in 1923 [9], the sex-determining loci have been narrowed down by the construction of the deletion-based maps to identify SPF and GSF [10,11]. Recently, these studies have moved forward with large-scale sequencing, leading to the specification of candidate genes for SPF and GSF, as well as *Microbotryum* SPF-like factor [12]. A further question relates to how they play roles in the sexual development of a dioecious plant. An answer to this question has been awaiting further development of functional analyses that could be used in *S. latifolia* plants. One of the difficulties in performing functional analyses is the resistance to *Agrobacterium*, because only limited ecotypes of *S. latifolia* plants are susceptible to a limited number of *Agrobacterium* strains. In fact, a recently developed *Agrobacterium*-mediated transformation method is now available for a specific *S. latifolia* ecotype, with an efficiency of up to 4.7% [13]. An alternative method that does not rely on *Agrobacterium* infection may solve the problem.

In this study, we developed a virus vector as a functional analysis system in *S. latifolia* plants. Plant virus vectors are used as versatile reverse-genetic tools for efficient functional analysis [14,15,16,17]. Since they can heterologously express a gene in plants, or can repress expression of a gene by virus-induced gene silencing (VIGS), the method can be applied for plants that are not susceptible to *Agrobacterium*. However, there are drawbacks that are associated with this system; virus vectors often retain pathogenic characters, such as symptoms induced by the virus itself, and they have a limited host range and tissue specificity. A virus used in this study, *Apple latent spherical virus* (ALSV), has advantages for analyzing flower morphology because it systemically infects plants, including floral tissues, without any symptoms [18]. Therefore, we developed the VIGS system using ALSV in *S. latifolia* plants. Our results demonstrated that ALSV infected *S. latifolia* plants at a rate of up to 100%, the infected plants had no symptoms, and virus vectors were detected throughout the flower meristems. VIGS was confirmed by knockdown of the phytoene desaturase gene of *S. latifolia* plants, and this was further applied to the floral organ genes, *SlUFO* and *SlSUP*, to observe the resulting phenotype in flowers. 

## 2. Results and Discussion

### 2.1. Optimization of an Inoculation Method for Delivering Apple Latent Spherical Virus (ALSV) to S. latifolia Plants

**Figure 1:**
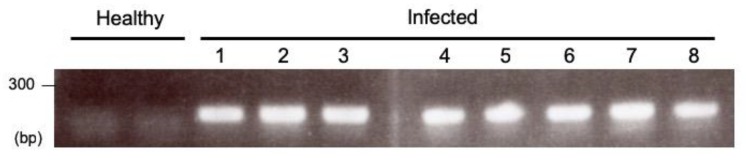
Reverse transcription polymerase chain reaction (RT-PCR) detection of *Apple latent spherical virus* (ALSV) from the upper leaves of *Silene latifolia* plants at 21 days post-inoculation (dpi). Lane 1–4, one-shot inoculation was applied to the plants; lane 5–8, two-shot inoculation to the plants. Two healthy plants were used as uninfected healthy controls.

*Apple latent spherical virus* (ALSV) has favorable characteristics as a virus vector, since it infects a variety of plant species, including legume, cucurbits, and Rosaceae fruit trees, and that its infection is asymptomatically and systemically dispersed throughout the plant, including the floral organs [18]. Based on these characteristics, it is likely that ALSV infects *S. latifolia* plants. To test this, we first inoculated ALSV in *S. latifolia* plants using a simple *Agrobacterium*-mediated inoculation method. However, we could not find any infection in *S. latifolia* plants, probably due to their resistance to *Agrobacterium*. We therefore took additional steps prior to the inoculation of *S. latifolia* plants, using *Nicotiana benthamiana* plants to avoid direct *Agrobacterium* inoculation onto *S. latifolia* plants. ALSV was propagated in *N. benthamiana* plants, and the middle leaves—which contained the highly infectious ALSV [19]—were collected to extract virus particles. Extracted RNA from the virus particles was applied to *S. latifolia* seedlings using a gene gun. This resulted in an infection rate that reached almost 100% (Figure 1). 

### 2.2. ALSV Migrates to Flowers in S. latifolia Plants

**Figure 2:**
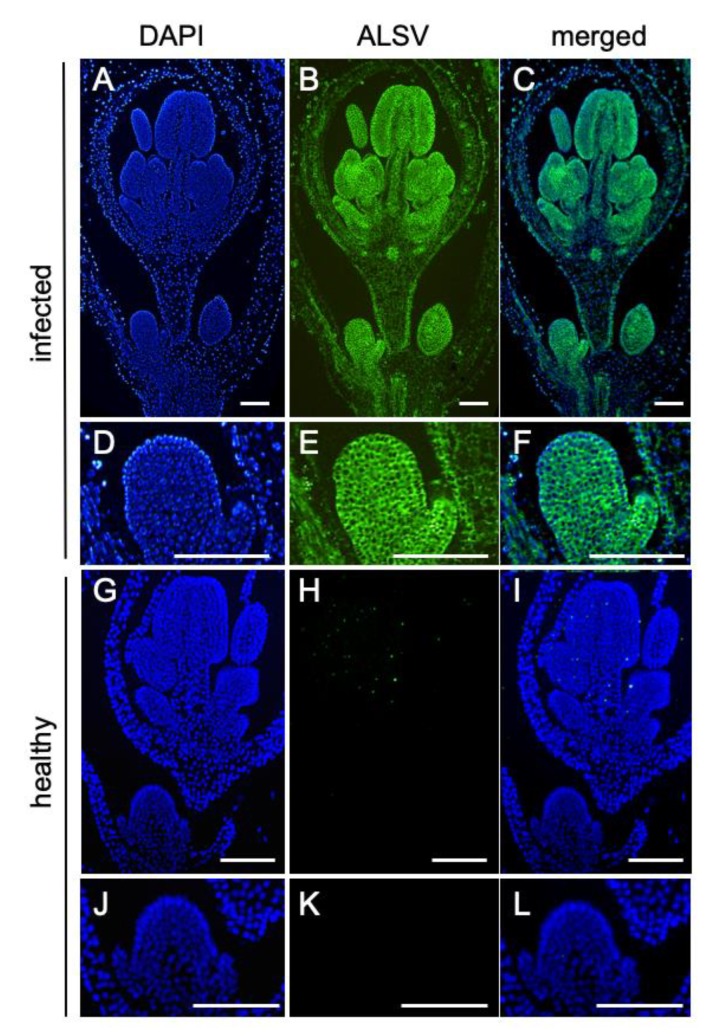
ALSV localization in floral buds of *S. latifolia* plants. (**A**–**F**) Flower buds infected with ALSV and (**G**–**L**) uninfected healthy flower buds. (**A**,**D**,**G**,**J**) Nuclei stained with 4′,6-diamidino-2-phenylindole (DAPI), (**B**,**E**,**H**,**K**) ALSV, detected by Fluorescein-5-isothiocyanate (FITC), and (**C**,**F**,**I**,**L**) their merged images. (**A**–**C**) Infected flower buds at a late stage and (**D**–**F**) enlarged images of the infected flower buds at an early stage. (**G**–**I**) Healthy flower buds at an early stage and (**J**–**L**) enlarged images of the healthy flower buds at an early stage. Representative images of at least three individual plants are shown. Bars = 100 µm.

The shoot apical meristem (SAM) is generally a virus-free region; however, several viruses, including *Turnip mosaic virus*, *Barley stripe mosaic virus*, and ALSV, can infect the SAM [20,21,22]. To evaluate whether ALSV migrates to the SAM and, subsequently, to the flowers, we performed in situ hybridization of *S. latifolia* floral buds infected with ALSV (Figure 2). The result suggested that ALSV migrates to the shoot apex, and that it persisted throughout the floral organs from the initial (Figure 2E,F) to subsequent stages (Figure 2B,C) of flower development. Importantly, no symptoms developed in the infected plants, while ALSV was dispersed throughout the floral meristems. These characteristics of ALSV are particularly useful for functional analyses in flowers, such as for studying sex determination. 

### 2.3. Evaluation of Virus-Induced Gene Silencing (VIGS)-Mediated Gene Knockdown in S. latifolia Plants 

**Figure 3:**
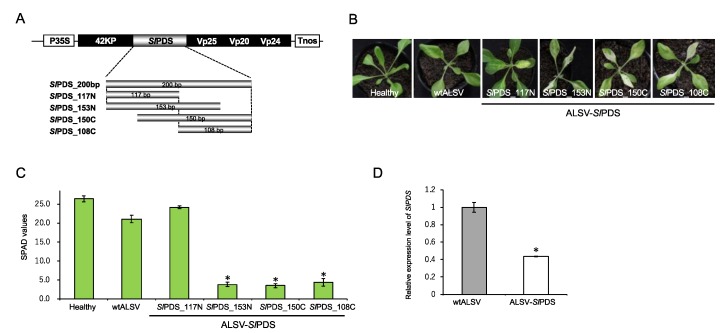
Virus-induced gene silencing (VIGS) of *SlPDS* gene in *S. latifolia* plants. (**A**) Schematic representation of ALSV-*Sl*PDS vectors used for VIGS. Four partial *SlPDS* fragments shown below, which differ in positions and length, were inserted into the artificial processing site of ALSV vector; (**B**) photographs of *S. latifolia* plants infected with ALSV-*Sl*PDS vectors at 20 dpi. Healthy represents a *S. latifolia* plant without any virus infection, and wtALSV represents a *S. latifolia* plant infected with wild-type ALSV vector without an *SlPDS* insert; (**C**) relative chlorophyll contents measured by the Soil Plant Analysis Development (SPAD) values in leaves infected with wtALSV and ALSV-*Sl*PDS vectors. SPAD values represent mean ± SE from three leaves of each plant (*n* = 3 to 7). Asterisks indicate a significant difference between plants infected with wtALSV and those infected with each ALSV-*Sl*PDS vector at *p* < 0.001 (Student’s *t*-test); (**D**) quantitative reverse transcription PCR (RT-qPCR) analysis of *SlPDS* gene expression levels in plants infected with wtALSV, and those infected with ALSV-*Sl*PDS. The error bars represent the standard deviations of at least three plants normalized to the *S. latifolia* 18S ribosomal RNA gene. The asterisk indicates a significant difference between plants infected with wtALSV, and those infected with ALSV-*Sl*PDS at *p* < 0.001 (Student’s *t*-test).

To validate whether the ALSV vector can reduce the expression of an endogene of *S. latifolia*, we used the phytoene desaturase (*SlPDS*) gene as an indicator of gene knockdown. *PDS* has been widely used for gene knockdown studies because its knockdown phenotype can be simply observed as photobleaching [23]. To construct ALSV-*Sl*PDS, we used a partial complementary DNA (cDNA) fragment of the *SlPDS* gene, obtained from total RNA of *S. latifolia* leaves, and cloned four sub-fragments of *SlPDS* into the ALSV vector, the length of which were 108, 117, 150, and 153 bp (Figure 3A, see also Section 3). The *Sl*PDS inserts were stably retained during the infection of these ALSV-*Sl*PDS vectors in *S. latifolia* plants, which was confirmed by reverse transcription polymerase chain reaction (RT-PCR). The plants inoculated with ALSV-*Sl*PDS vectors exhibited photobleaching phenotypes that were typical for *PDS* knockdown within 20 days after inoculation, indicating that the VIGS system worked efficiently in *S. latifolia* plants (Figure 3B). 

The extent of the photobleaching was slightly different between the four ALSV-*Sl*PDS constructs, suggesting that the sequence length and content of the insert affect VIGS efficiency. The insert length was especially important, because inserts with more than 200 bp were all deleted on the inoculated or upper leaves; hence, VIGS was unsuccessful. This result was consistent with a previous study that showed that a shorter insert tends to be more stable than a longer one [18]. The photobleaching phenotype is especially prominent along the vasculature of each leaf, which is likely to reflect the higher level of virus accumulation in that tissue. To quantify the bleaching phenotypes, we analyzed SPAD values in each bleached leaf, which are known to be proportional to the amount of chlorophyll content in the leaves [18]. An analysis of SPAD values indicated that they were decreased when leaves exhibited a photobleaching phenotype upon ALSV-*Sl*PDS infection (Figure 3C). While the SPAD values of plants infected with ALSV-*Sl*PDS_117N were almost similar to those of the wild-type ALSV (wtALSV) and of uninfected plants, the SPAD values of plants infected with ALSV-*Sl*PDS_153N, _150C, and _108C were significantly lower than those of uninfected plants (*p* < 0.05, Student’s *t*-test). The knockdown of the *SlPDS* gene was confirmed by RT-qPCR analysis (Figure 2D). Taken together, the results demonstrated that VIGS-mediated gene knockdown by ALSV vectors can decrease *S. latifolia* gene expression. 

**Figure 4:**
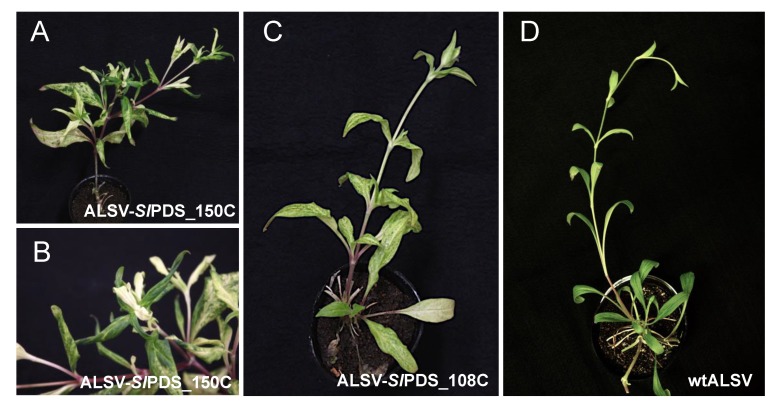
(**A**) Systemic phenotype of the plant infected with ALSV-*Sl*PDS_150C and (**B**) enlarged image of the shoot apex in (**A**). (**C**) The plant infected with ALSV-*Sl*PDS_108C and (**D**) wild-type ALSV. Bars = 10 cm (single line), 2 cm (double line).

To examine the persistence of the ALSV vector in *S. latifolia* plants, we continued to observe the phenotypes of the ALSV-*Sl*PDS infected plants for about two months. As a result, the photobleaching phenotype remained and was distributed systemically, but in a patchy fashion in the lower and middle leaves; homogeneous bleaching was observed in the upper leaves (Figure 4). The result indicates that ALSV accumulates in the upper parts of the plant, as previously reported in other plants [18]. This accumulation in the upper parts is typical in ALSV-infected plants, which is particularly advantageous for the functional analysis of floral organ genes. 

### 2.4. VIGS-Mediated Gene Knockdown in the Flowers of S. latifolia Plants

We then applied the developed ALSV system to study gene function in the flowers of *S. latifolia* plants. Two genes, *SlUFO* and *SlSUP*, a homolog of *Arabidopsis UNUSUAL FLORAL ORGANS* (*UFO*) and *SUPERMAN* (*SUP*), respectively, were used for the assay. UFO is an F-box protein, which acts as a cofactor for LEAFY (LFY) transcription factor in the flower primordia [24]. LFY, together with APETALA1 (AP1) plays a role in the specification of flower meristem identity [25,26,27,28]. In *S. latifolia*, *SlUFO* is expressed at the base of the petal primordia during the last stages of flower development (Figures S2 and S3). Recent studies have shown that the activity of the Class I KNOTTED-like homeobox (KNOXI) transcription factor SHOOT MERISTEMLESS (STM) affects *UFO* expression [29], therefore, it could be expected that VIGS-mediated *SlUFO* knockdown caused drastic changes in floral architecture. *SlSUP* is exclusively expressed in female flower buds; overexpression of *SlSUP* in hermaphrodite *A. thaliana* plants resulted in suppression of stamens [30]. It has been shown that the expression level of *SlSUP* was significantly decreased in the female flowers, exhibiting a hermaphroditic phenotype due to *Microbotryum* infection [30] (the *Microbotryum*-infected phenotype is shown in the cover figure). The known data, therefore, suggested that *SlSUP* gene knockdown in a *S. latifolia* female flowers would produce a hermaphroditic phenotype. 

**Figure 5:**
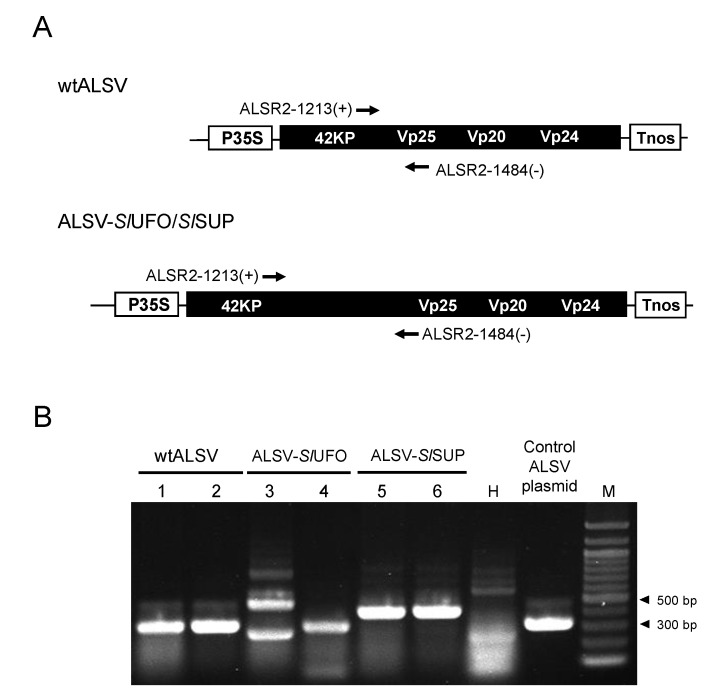
Verification of ALSV infection and the retention of the insert in ALSV-*Sl*UFO and -*Sl*SUP plants. (**A**) Schematic representation of wtALSV and ALSV-*Sl*UFO/*Sl*SUP. The insert size is 162 and 132 bp for ALSV-*Sl*UFO and *Sl*SUP, respectively. Arrows indicate a primer set flanking the insert used for RT-PCR; (**B**) representative result of the RT-PCR in the flower buds inoculated with wtALSV (Lane 1 and 2), ALSV-*Sl*UFO (Lane 3 and 4), and ALSV-*Sl*SUP (Lane 5 and 6). The size of the amplification products from wtALSV, which has no insert, is approximately 300 bp. The retention of the insert was determined by the fragment length. H, uninfected healthy plant; control ALSV plasmid, the ALSV plasmid without the insert; M, 100 bp ladder marker.

To induce *SlUFO*/*SlSUP* knockdown in *S. latifolia* flowers, we generated ALSV-*Sl*UFO and ALSV-*Sl*SUP, and introduced them to *S. latifolia* plants, as described above. The inserts finally used for ALSV-*Sl*UFO and ALSV-*Sl*SUP were 162 and 132 bp in length, respectively. These constructs were selected from three *Sl*UFO (132, 162, and 174 bp in length) and five *Sl*SUP (102, 105, 111, 132, and 177 bp in length) constructs, based on the infection rate to *S. latifolia* (see Table S1 primer list for details of each fragment position), and they were used for further analysis. We checked the infection of ALSV vector carrying the *Sl*UFO or *Sl*SUP insert in each plant by RT-PCR, using primers flanking the insert (Figure 5A), both from the upper leaves and the floral organs. The results showed that ALSV-*Sl*UFO and ALSV-*Sl*SUP infected *S. latifolia* plants at high rates in the upper leaves: up to 100% and 60% for ALSV-*Sl*UFO and ALSV-*Sl*SUP, respectively. In addition, the retention of the *Sl*UFO or *Sl*SUP insert was verified in all plants that were infected by ALSV in the upper leaves, demonstrating that ALSV-*Sl*UFO and -*Sl*SUP were successfully delivered to *S. latifolia* plants. In the floral organs, however, we found a high rate of deletion of the *Sl*UFO or *Sl*SUP insert: 50%–100% and 33%–67% deletion for ALSV-*Sl*UFO and ALSV-*Sl*SUP, respectively, depending on the inoculation conditions (gas pressure). Figure 5 shows the representative results of RT-PCR from ALSV-infected plants. RT-PCR from flower buds of ALSV-*Sl*UFO-inoculated plants showed two patterns, multi-bands, or a single band. The multi-bands indicated that the flower buds were co-infected with the vector retaining the insert, as well as with that from which the insert was deleted (Lane 3, Figure 5B), whereas the single band indicated that these buds were only infected with the vector from which the insert was deleted (Lane 4, Figure 5B). By contrast, the vector retaining the *Sl*SUP insert was often detected as a single band (Lanes 5 and 6, Figure 5B), indicating that the vector stability in the flower buds was relatively high in the ALSV-*Sl*SUP-inoculated plants. 

**Figure 6:**
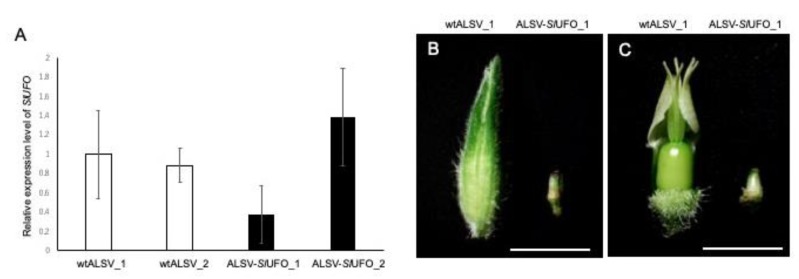
Relative expression level of the *SlUFO* gene and the exhibited phenotype in ALSV-*Sl*UFO plants. (**A**) RT-qPCR analysis of *SlUFO* gene expression levels in plants infected with wtALSV and ALSV-*Sl*UFO. A bud smaller than 2 mm was collected from different individual plants. Values represent means ± standard deviations of two technical replicates normalized to *S. latifolia ubiquitin 11* gene; (**B**) a representative phenotype observed in a bud infected with wtALSV (**left**) and ALSV-*Sl*UFO (**right**). The buds shown here are derived from the same plants used for the RT-qPCR in (**A**). The bud size of the ALSV-*Sl*UFO-infected plant is relatively small, compared with that of the wtALSV-infected plant; (**C**) pictures taken after removal of the sepals from (**B**). Despite their small size, all floral organs appear normal. Bars = 1 cm.

Next, we analyzed whether the expression of *SlUFO* was downregulated in ALSV-*Sl*UFO-infected flower buds. *SlUFO* expression was decreased in the buds infected with the ALSV-*Sl*UFO vector that partially retained the insert (Lane 3 in Figure 5B), although they were not statistically significant (Figure 6A, ALSV-*Sl*UFO_1), suggesting that ALSV-*Sl*UFO caused VIGS in the floral organs. By contrast, the bud infected with ALSV, from which the *Sl*UFO insert was completely deleted (like lane 4 in Figure 5B), showed an even higher level of *SlUFO* expression (Figure 6A, ALSV-*Sl*UFO_2). From these observations, we concluded that the decrease in *SlUFO* expression was only prominent in buds infected with ALSV-*Sl*UFO that retained the insert. The phenotype observed in those buds was mild; they were reduced in size and failed to open up (Figure 6B). Similar phenotypes have been reported in a *ufo* mutant of *Torenia* plants, in which the second whorl was transformed to sepal-like organs [31]. However, when we removed its sepals, we observed a normal structure of floral organs in ALSV-*Sl*UFO plants (Figure 6C), unlike the sepaloid phenotype in *Torenia* plants [31]. Since *UFO* is known to play roles in flower development in concert with crucial transcription factors for flowering initiation [24,25,26,27,28,29], our result suggested that knockdown of the *SlUFO* gene caused the inhibition of flower development at the initial stages. 

**Figure 7:**
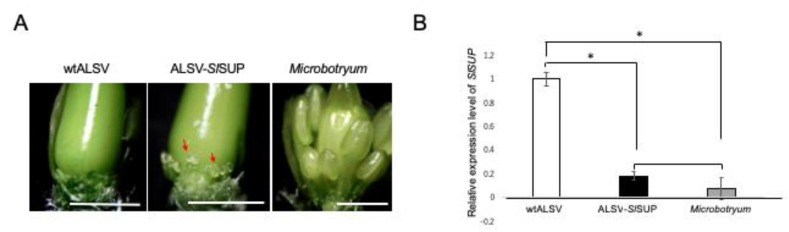
Downregulation of the *SlSUP* gene by VIGS in *S. latifolia* plants. (**A**) Different levels of stamen induction in female *S. latifolia* infected with wtALSV, ALSV-*Sl*SUP, and *Microbotryum*. Stamens are not developed in female flowers in the wtALSV plants, while marginally and fully developed stamens are observed in female flowers in ALSV-*Sl*SUP- and *Microbotryum*-infected plants, respectively. Red arrows indicate the stamens in ALSV-*Sl*SUP plants. Bars = 1 mm; (**B**) RT-qPCR analysis of *SlSUP* gene expression levels in the female flower meristems infected with wtALSV, ALSV-*Sl*SUP, and *Microbotryum*. Five flower meristems corresponding to stage 7–10 were collected, and the stamen induction was checked under a microscope in ALSV-*Sl*SUP- and *Microbotryum*-infected plants. Two out of five buds had confirmed stamen induction in ALSV-*Sl*SUP plants. All buds had confirmed stamen induction in *Microbotryum*-infected plants. The error bars represent the standard deviations at those two buds, normalized to the *S. latifolia ubiquitin 11* gene. Asterisks indicate a significant difference; there was no significant difference between plants infected with ALSV-*Sl*SUP and *Microbotryum* at *p* < 0.05 (Student’s *t*-test).

In ALSV-*Sl*SUP plants, we observed stamen induction in the female flowers, which was weak compared to the hermaphroditic phenotype induced by *Microbotryum* infection (Figure 7A). To assess the contribution of *SlSUP* expression level to the phenotype, we compared the *SlSUP* gene expression level between ALSV-*Sl*SUP- and *Microbotryum*-infected plants. The expression level of *SlSUP* was significantly decreased in the flower primordial, compared with that in the plants infected with wtALSV (Figure 7B; *p* < 0.05, Student’s *t*-test), which was of a similar level (*p* > 0.05, Student’s *t*-test) to that observed in the *Microbotryum*-infected plants. The results showed that the expression of the *SlSUP* gene was successfully downregulated by ALSV-mediated VIGS, but that this was not enough to cause complete stamen induction, similarly to the *Microbotryum*-mediated hermaphroditic formation. 

## 3. Materials and Methods

### 3.1. Plant and Viral Materials, and Agroinfiltration

*Silene latifolia* seeds used in this study were originally collected from a field population in Berlin, Germany, or a generous gift from Professor Michael E. Hood (Amherst College, Amherst, MA, USA). *S. latifolia* and *N. benthamiana* plants were grown in a growth chamber under conditions of 25 °C with a daylength of 16 h. The methods used for *Agrobacterium tumefaciens* infiltration were previously described [32]. *Agrobacterium* cultures carrying an appropriate plasmid were resuspended in infiltration buffer to a final optical density of 1.0 at 600 nm. 

### 3.2. Inoculation of ALSV onto S. latifolia Plants 

Methods for the inoculation of ALSV vector into *S. latifolia* plants were performed according to a recently updated protocol [19]. Briefly, *Agrobacterium* cultures containing pCAMBIA1301-ALSV-RNA1, pCAMBIA1301-ALSV-RNA2 without/with a target fragment, and RNA silencing suppressor p19 of tomato bushy stunt virus, were mixed at a 1:1:1 ratio and infiltrated into three leaves of 3- to 4-week-old *N. benthamiana* plants. p19 was added to increase the virus infectivity, which was only applied upon inoculation, to *N. benthamiana* plants. After confirming ALSV infection and the retention of the insert in the upper uninoculated leaves two weeks after inoculation, the infected *N. benthamiana* leaves were processed to concentrate virus particles, using bentonite solution (the detailed procedure is described in [19]). Virus RNA was extracted from the concentrated virus solution, using phenol extraction and ethanol precipitation, and 250 µg of each RNA was mixed with gold particles (Microcarrier, Bio-Rad Laboratories, Hercules, CA, USA). This RNA-coated gold was inoculated into 2-week-old *S. latifolia* plants using the PDS-1000/He Particle Delivery System (Bio-Rad) or GDS-80 (Nepa Gene Co., Ltd., Chiba, Japan).

### 3.3. RNA Extraction and Reverse Transcription Polymerase Chain Reaction (RT-PCR) for Checking ALSV Infection

Total RNA was extracted from *S. latifolia* leaves by using the ISOGEN reagent (FUJIFILM Wako Chemicals, Osaka, Japan) or TRIzol reagent (Thermo Fisher Scientific, Waltham, MA, USA). One-step RT-PCR was performed by using the SuperScript III One-Step RT-PCR System with Platinum Taq (Thermo Fisher Scientific). The primer set R2ALS1363(+) and R2ALS1551(−) was used for detecting ALSV (Figure 1). The primer set ALSR2-1213(+) and ALSR2-1484(−), which was designed to flank the multiple cloning site of the ALSV vector, was used for detecting ALSV in inoculated plants, and for checking whether the infected vector retained the insert. The reaction volume was 10 µL, and the thermal cycling conditions were 50 °C for 30 min for reverse transcription, 94 °C for 2 min to activate the DNA polymerase; followed by 40 cycles of denaturation at 94 °C for 15 s, annealing at 55 °C for 30 s, and extension at 68 °C for 1 min; with a final extension at 68 °C for 7 min.

### 3.4. In Situ Hybridization Analysis to Detect Virus Invasion into the Floral Meristems

Shoot apices of ALSV-infected plants were sampled and fixed in 4% paraformaldehyde. Tissue sections were prepared by Kawamoto’s film method [33]. RNA in situ hybridization was performed by using the methods described in [34]. A digoxigenin (DIG)-labeled antisense RNA probe, that was complementary to positions 1418 to 2111 of ALSV-RNA2, was used for the detection of ALSV. Anti-DIG-FITC (Sigma-Aldrich, St. Louis, MO, USA) was used for signal detection.

### 3.5. Construction of ALSV Vectors

ALSV vectors used in this study were based on pCAMBIA1301-ALSV-RNA1 and pCAMBIA1301-ALSV-RNA2, a T-DNA-based plasmid containing the full-length cDNA clone of pEALSR1 and pEALSR2L5R5, respectively [17]. pEALSR2L5R5 was modified to have a multiple cloning site containing *Sal*I, *Bln*I, *Aor51*HI, and *Bam*HI, to make unique restriction enzyme sites in the pCAMBIA1301-based vector. Primers used in this study are listed in Table S1. For the construction of ALSV-*Sl*PDS, a partial cDNA of *SlPDS* was amplified from DNase-treated total RNA of *S. latifolia* by reverse-transcription with PrimeScript RTase (Takara Bio, Otsu, Shiga, Japan), and subsequent PCR with KOD-FX Neo (Toyobo, Osaka, Japan) using primer set atPDS_1070F and atPDS_1809R, which were designed based on the *AtPDS* gene sequence of *A. thaliana*. Subsequently, four fragments of *Sl*PDS were amplified from this cDNA using four primer sets: SlPDS-100F and SlPDS-200R for SlPDS_100N (the length of the amplified product was 117 bp), SlPDS-100F and SlPDS-250R for SlPDS_150N (153 bp), SlPDS-150F and SlPDS-300R for SlPDS_150C (150 bp), and SlPDS-200F and SlPDS-300R for SlPDS_100C (108 bp). For the construction of ALSV-*Sl*UFO and ALSV-*Sl*SUP, the partial cDNAs of *SlUFO* (Figure S1; GenBank accession No. MK563991) and *SlSUP* [30] were used as a template for amplification with primer sets ALSV-sal-SlUFO_1029F and ALSV-bam-SlUFO_1190R, and ALSV-sal-SlSUP_310F and ALSV-bam-SlSUP_441R, respectively. All PCR-amplified fragments were excised from 3% agarose gels, purified, and cloned into *Sal*I- and *Bam*HI-treated pCAMBIA1301-ALSV-RNA2 using the Gibson Assembly Cloning Kit (New England Biolabs, Ipswich, MA, USA). *Sl*PDS-, *Sl*UFO- or *Sl*SUP- cloned ALSV vectors were verified by sequencing with ALSV-2600F and ALSV-3000R, and transformed into an *Agrobacterium* strain, EHA105, using the freeze-thaw method [35].

### 3.6. RT-PCR and Quantitative RT-PCR (RT-qPCR)

Quantitative RT-PCR (RT-qPCR) was carried out as previously described [32]. Primers for detecting the *SlPDS* gene were SlPDSF1 and SlPDSF2; for the *SlUFO* gene, SlUFO_F2 and SlUFO_R2; for the *SlSUP* gene, SlSUPF4 and SlSUPR3 [19]. These qPCR primers were designed in the regions which are different from the VIGS insert region. The primers for 18S ribosomal RNA (rRNA) gene, Sl18SF1 and Sl18SR1 [30], and *ubiquitin 11* gene, SL_UBQ F and SL_UBQ R [36], were used as references.

### 3.7. Inoculation of Microbotryum onto S. latifolia Plants

*Microbotryum* strains (ATCC-22000 and -22004, [8]) were inoculated onto *S. latifolia* plants as previously described [37], with an infection rate of approximately 80%–90%.

## 4. Conclusions

This is the first report of a virus vector system applied to *S. latifolia* plants. Our results demonstrated that the broad host range of ALSV could be extended to *S. latifolia* plants. The developed method has advantages for functional analysis in *S. latifolia* plants, because the ALSV vector can be delivered to plants at high rates of up to 100%, and it asymptomatically and systemically infects plant tissues, including the floral organs. Taking advantage of this high gene delivery rate and the ability of ALSV to invade the meristematic tissue, it may be applicable to develop novel and more stable functional analysis methods, such as transformation or genome editing, like the recently reported genome editing using a virus vector [38]. Collectively, this study showed that the ALSV vector can cause target gene knockdown of *S. latifolia* plants by VIGS, and this technique will allow for unveiling of gene functions and networks of *S. latifolia* plants, such as the mechanisms of sex determination and fungal-induced masculinization.

## Supplementary Materials

Supplementary materials can be found at https://www.mdpi.com/1422-0067/20/5/1031/s1.
