# Supplementary material for: Development of the VIGS System in the Dioecious Plant Silene latifolia
Source: Int J Mol Sci. 2019 Feb 27;20(5):1031. doi: 10.3390/ijms20051031 (PMC6429067; doi:10.3390/ijms20051031)
Supplement: Supplementary file 1 [file ijms-20-01031-s001.pdf]

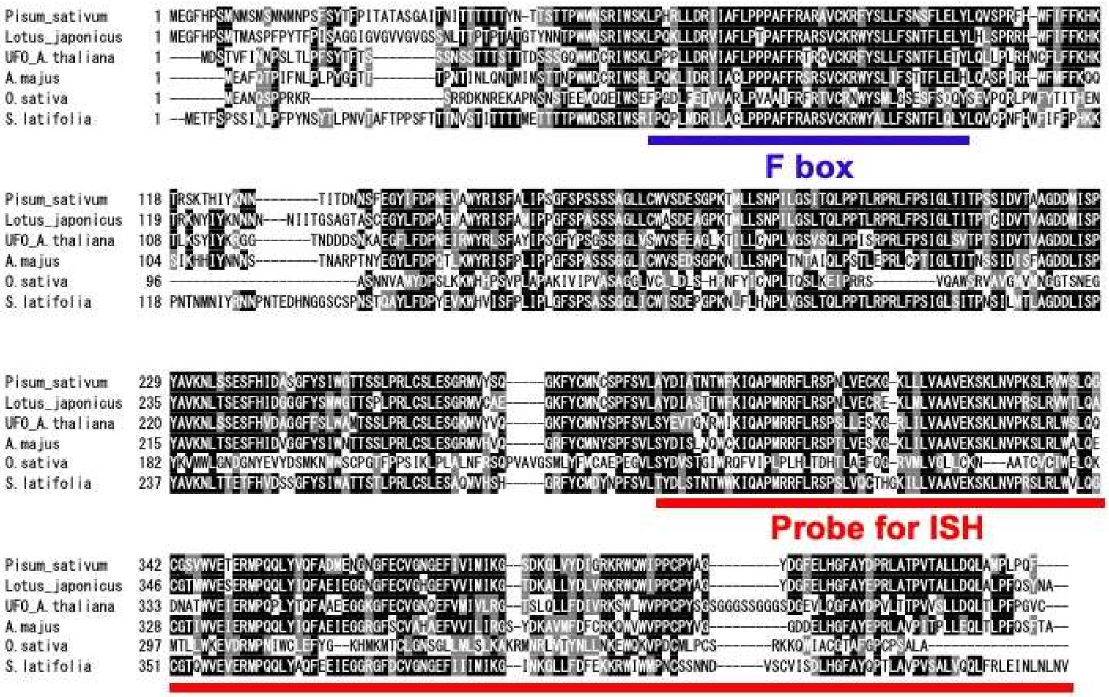

Figure S1. SlUFO amino acid sequence and alignment of its homolog genes in other plant species. The highlighted square indicates a homologous amino acid conserved among UFOs. The F-box and probe region for in situ hybridization (ISH) are indicated with blue and red line, respectively.

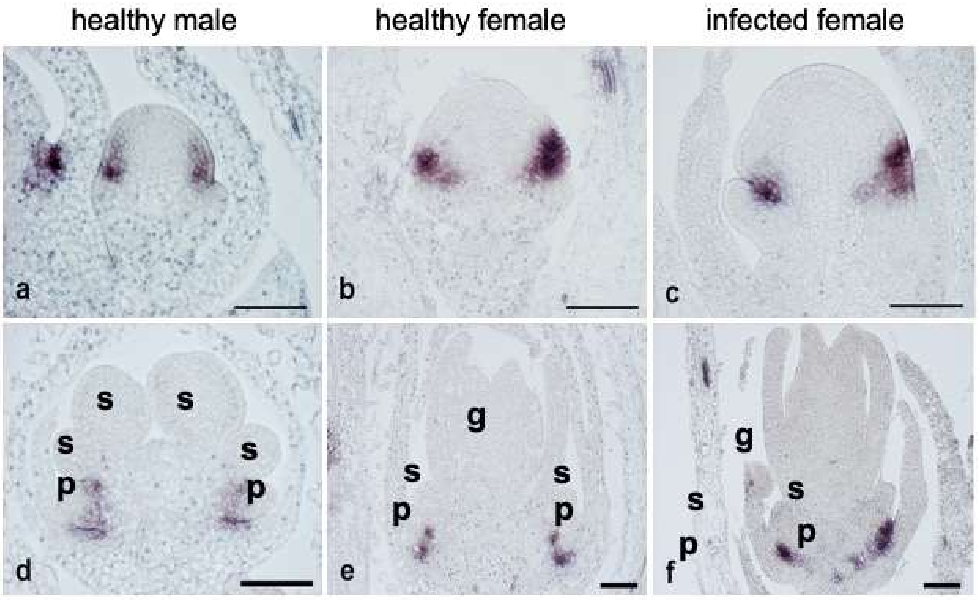

Figure S2. In situ hybridization analysis of *SlUFO* mRNA in *S. latifolia* flowers at an early flower development stage. (**a**) Healthy male flower, (**b**) healthy female flower, and (**c**) *Microbotryum* infected female flower at stage 2; (**d**) Healthy male flower at stage 8 (**e**) healthy female flower and (**f**) *Microbotryum* infected female flower at stage 7. g, gynoecium; p, petal; s, stamen. Bars = 100 μm.

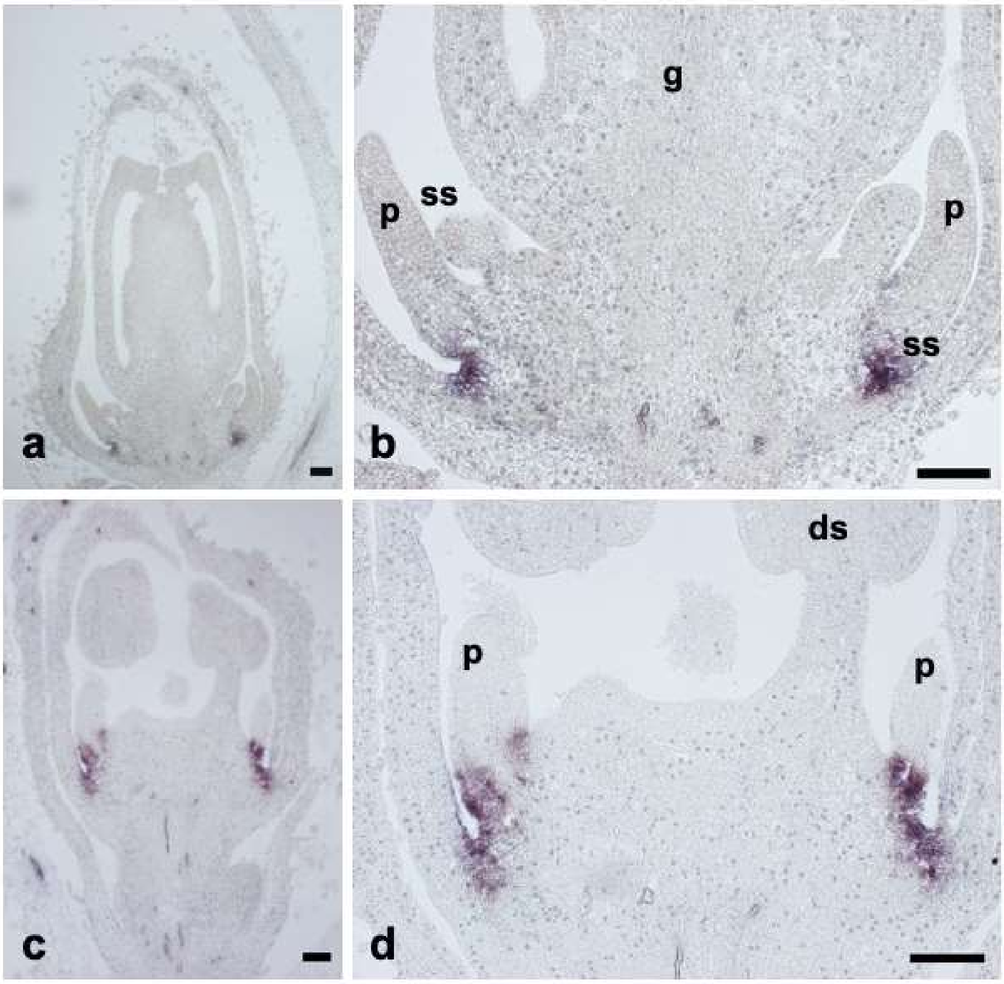

Figure S3. In situ hybridization analysis of *SlUFO* mRNA in *S. latifolia* flowers at a late flower development stage. (**a**) Female flower at stage 12 and (**b**) higher magnification. (**c**) Hermaphroditic flower resulted from *Microbotryum* infection at stage 12, and (**d**) higher magnification. g, gynoecium; p, petal; ds, developing stamen; ss, suppressed stamen. Bars = 100 μm.

Table 1. List of oligonucleotide primers used in this study.

Number	Name	Sequence (5′-3′)	Reference
1	R2ALS1363(+)	GCGAGGCACTCCTTA	ALSV manual
2	R2ALS1551(-)	GCAAGGTGGTCGTGA	ALSV manual
3	ALSV-2600F	AGTGCTTCCACTCGTTTTAC	This study
4	ALSV-3000R	GTAGCATGACCACTCATGAT	This study
5	ALSR2-1213(+)	ATACCACCTCATACAGGTACAC	Kasajima et al. 2017
6	ALSR2-1484(-)	CGTTCCACGACCGTGTGGCCAGA	Kasajima et al. 2017
7	SlPDSF1	ACCCGAGGAATGGAAAGAGA	This study
8	SlPDSF2	TGCTGAAAAGTAGATGGTCGTATGT	This study
9	SlUFO_F2	CAATGCACACATGGCAAAAT	This study
10	SlUFO_R2	CAAACCCTCTTCCTCCTTCA	This study
11	SlSUPF4	CCG ACG ATC ATG GAA TCA TCA AGA	Kazama et al. 2009
12	SlSUPR3	CCA AGA CGA AGC TCC AAA TCC A	Kazama et al. 2009
13	Sl18SF1	GGC AAC GGA TAT CTC GGC TCT C	Kazama et al. 2009
14	Sl18SR1	TGA CGC CCA GGC AGA CGT GC	Kazama et al. 2009
15	SL_UBQ F	AATTTTCGCCTTCCTCATCC	Zemp et al. 2014
16	SL_UBQ R	GCTTGCCAGCGAAAATAAGT	Zemp et al, 2014
17	atPDS_1070F	ACCGACGAGGTGTTTATTGG	This study
18	atPDS_1809R	CGACATGGTTCACAGTTTGG	This study
19	SlPDS-100F	GGCCCTGATTTCACAgtcgacCAGGAGAAGCATGGTTCC	This study
20	SlPDS-200R	CCCTGACCTTCTAGCAGggatccACGAGAGTTAAGCTTCACTTCACC	This study
21	SlPDS-250R	CCCTGACCTTCTAGCAGggatccCACACTTCCATCCTCGTTCAACTC	This study
22	SlPDS-150F	GGCCCTGATTTCACAgtcgacAGGCTGTGCATGCCCATTG	This study
23	SlPDS-300R	CCCTGACCTTCTAGCAGggatccATAAGCATCTCCTCTTATCTCTTTTC	This study
24	SlPDS-200F	GGCCCTGATTTCACAgtcgacGGTGAAGTGAAGCTTAACTCTCGT	This study
25	ALSV-sal-SlUFO_1029F	GGCCCTGATTTCACAgtcgacACATGGTGGAAAATCCAAGC	This study
26	ALSV-sal-SlUFO_1159F	GGCCCTGATTTCACAgtcgacGATTGTGGGTCCTACAAGGATG	This study
27	ALSV-sal-SlUFO_1245F	GGCCCTGATTTCACAgtcgacGAAGGAGGAAGAGGGTTTG	This study
28	ALSV-bam-SlUFO_1190R	CCCTGACCTTCTAGCAGggatccTTGGGTCCCACATCCTTGTAG	This study
29	ALSV-bam-SlUFO_1290R	CCCTGACCTTCTAGCAGggatcc TAATAAACTCACCATTTCCAACAC	This study

30	ALSV-bam-SlUFO_1418R	CCCTGACCTTCTAGCAGggatccTGCAAATCCATGCAAATCA	This study
31	ALSV-sal-SlSUP_310F	GGCCCTGATTTCACAgtcgacCCAAACCCTAACCCTAACCCT	This study
32	ALSV-sal-SlSUP_331F	GGCCCTGATTTCACAgtcgacAATCCAAACCCTAGCTCAAATCTT	This study
33	ALSV-sal-SlSUP_406F	GGCCCTGATTTCACAgtcgacTCACTCTCACTTTCCTCTCTTCCA	This study
34	ALSV-bam-SlSUP_441R	CCCTGACCTTCTAGCAGggatccCAATGGAGGTGGTGGAAGAGA	This study
35	ALSV-bam-SlSUP_507R	CCCTGACCTTCTAGCAGggatccTTTCCATTTCCCTATCCCATC	This study

Materials and Methods

Isolation of SlUFO

SlUFO partial sequence was obtained from cDNA prepared from male and female total RNA flower buds. A genomic DNA fragment and the complete coding region was obtained using above partial sequence.

In Situ Hybrdidization

Paraffin-sections of flower buds were prepared as described previously (Kazama et al. 2009). In situ hybridizations were performed using the probe shown in Figure 1. The procedure was applied accordingly to the previous report (Kazama et al. 2009).

Reference

1 Kazama, Y.; Fujiwara, M.T.; Koizumi, A.; Nishihara, K.; Nishiyama, R,; Kifune, E.; Abe, T.; Kawano, S. A SUPERMAN-like gene is exclusively expressed in female flowers of the dioecious plant *Silene latifolia*. *Plant Cell Physiol.* **2009**, *50*, 1127–1141.
